# Lidocaine Shows Significant Antimicrobial Effects Against *Staphylococcus* Species: An In-Vitro Study Comparing Different Combinations of Lidocaine and Clinically Used Injectables, like Steroids and Hyaluronan, in the Context of Arthritis Management

**DOI:** 10.3390/biomedicines13010106

**Published:** 2025-01-05

**Authors:** Stephan Heller, Ricarda Johanna Seemann, Rainer Burgkart, Andreas Obermeier, Hermann Locher

**Affiliations:** 1 Department of Orthopaedics and Sports Orthopaedics, TUM School of Medicine and Health, TUM Universitätsklinikum Klinikum Rechts der Isar, 81675 Munich, Germany; 2 Centre for Orthopaedics and Specialized Pain Therapy, 88069 Tettnang, Germany; 3 Charité–Universitätsmedizin Berlin, Corporate Member of Frei Universität Berlin und Humboldt-Universität zu Berlin, Centre for Musculoskeletal Surgery, 10117 Berlin, Germany

**Keywords:** lidocaine, septic arthritis, staphylococci, MRSA, periprosthetic joint infections

## Abstract

Introduction: Intra-articular injections, commonly used in osteoarthritis treatment, are debated due to their potential link to septic arthritis, though its incidence remains low. Lidocaine, used as a “carrier” for therapeutic substances like hyaluronan or triamcinolone, has pain-relieving and antimicrobial properties. This study investigates the concentration-dependent antimicrobial effects of lidocaine in combination with hyaluronan and triamcinolone in both standard and synovial fluid cultures. Methods: The antimicrobial efficacy of lidocaine against *Staphylococcus aureus* was investigated, with variations in bacterial and lidocaine concentrations. Bacterial growth was monitored using a UV/VIS spectrometer at 600 nm. Lidocaine solutions of 1% and 2% were tested, both alone and in combination with hyaluronic acid or Triam40, in tryptic soy broth (TSB), to reflect knee joint applications. The groups included pure lidocaine (L), Triam (T), hyaluronan (H), and combinations (LT, LH, TH, LTH) with 1% or 2% lidocaine. A bacterial inoculum of 300 CFU/mL was used, and samples were incubated for 12 and 24 h. Additional tests were conducted on *Staphylococcus epidermidis* and methicillin-resistant Staphylococcus aureus (MRSA), as well as on *S. aureus* in human synovial fluid. Results: Lidocaine showed a concentration-dependent antimicrobial effect, with greater inhibition at higher concentrations and lower bacterial densities. All lidocaine-containing combinations significantly reduced the bacterial levels of *S. aureus* in TSB. Similar results were seen for *S. epidermidis* and *MRSA*, with notable inhibition in synovial fluid after 12 h, especially with 2% lidocaine. Conclusions: Lidocaine exhibits dose-dependent antimicrobial effects against key pathogens responsible for septic arthritis. Its combination with Triam40 and hyaluronan may reduce the risk of septic arthritis, supporting its clinical relevance.

## 1. Introduction

*S. aureus* is the most common pathogen in septic arthritis, a potentially life-threatening disease that can lead to lasting damage to affected joints [1]. The incidence varies from 6 to 10 cases per 100,000 individuals per year [2]. It most frequently occurs in the knee and can be caused by the hematogenous spread of bacteria, by direct inoculation through wounds, or during medical procedures [3,4]. One frequently and controversially discussed risk factor for development of septic arthritis is previous intra-articular injections [3,4], routinely used, e.g., in standard conservative treatment of osteoarthritis. As known, the skin harbors up to 10^4^ to 10^6^ CFU/cm² [5]. Assuming a 20-gauge needle with a diameter of 0.9 mm and a resulting cross-sectional area of 0.0064 cm², this could result in an average potential transfer of approximately 600 pathogens per injection. This results in an average pathogen entry density of 100 CFU/mL due to the dilution effects from the injected solutions (e.g., 5 mL lidocaine, 2 mL hyaluronan, 1 mL Triam40). Consequently, a pathogen density of up to 100 CFU/mL within the knee is estimated in the case of insufficient disinfection.

Patients with altered joints have a much higher risk of developing septic arthritis than the average population. Osteoarthritis or degenerative joint disease is widespread in Western civilizations and presents a major burden in health economics today [6]. The prevalence has increased in recent decades and was over 6000 per 100,000 individuals in 2019 [7]. Therapeutic options encompass a range of both non-operative and operative interventions. It is widely accepted that conservative treatment methods should be thoroughly exhausted before considering surgical procedures such as total joint arthroplasty [8,9]. This includes the intra-articular injection of agents such as—among others—corticosteroids and hyaluronan [10]. The effect of corticosteroids is well documented for the short- and mid-term reduction in inflammation and pain in osteoarthritic joints [11,12], which is due to the inhibition of the anti-inflammatory cascade at various cellular levels in synovium and chondrocytes. The injection of substances like hyaluronan has been shown to have a positive effect on reducing pain, cartilage destruction, and subsequent bone damage [13,14].

While the isolated injection of local anesthetics (e.g., lidocaine) can be diagnostically valuable in determining whether the source of the perceived pain is located in the joint itself [15], it is common practice to use local anesthetics as “carriers” for the above-mentioned corticosteroids or hyaluronan. This combination offers therapeutic advantages: beyond providing temporary local anesthesia, local anesthetics such as lidocaine exhibit neurophysiological/physicochemical effects on the joint and its components. Additionally, they can down-regulate synovial nociceptors. It is assumed that the benefits of local anesthetic injections extend beyond their immediate effect; the rapid pain relief they provide can also help refunctionalize nociceptively altered motor control [16].

The antimicrobial effects of local anesthetics have been a topic of discussion for quite some time [17]. While some studies have found local anesthetics not to have antimicrobial effects, the majority of research indicates that lidocaine can inhibit or reduce the growth of numerous bacteria and fungi under various conditions [17,18,19]. However, there is little research specifically focused on its antimicrobial effects in the knee joint or on the interactions between lidocaine and synovial fluid or potential pathogens, which fundamentally requires further investigation in the future. Various antimicrobial mechanisms have been discussed in the literature and include damage to the cell membrane and thus the release of intracellular components, alteration of cellular respiration, and a reduction in enzyme activities [18,20,21,22]. The use of local anesthetics for intra-articular injections in the treatment of osteoarthritis remains controversial, as potential chondrotoxic effects have been described as well [23,24,25].

The aim of our study was to investigate the antimicrobial efficacy of different lidocaine concentrations, both as a standalone agent and in its conventional combination with hyaluronan (Ostenil^®^) and corticosteroids (triamcinolone, Triam^®^). When administering an injection, a skin-punching cylinder is always introduced into the knee joint, which can potentially also carry pathogens into the joint. Given the widespread prevalence of osteoarthritis, which often requires intra-articular injections as part of standard conservative treatment, one might expect a higher incidence of septic arthritis. However, the occurrence of septic arthritis remains relatively rare. This contrast naturally raises an intriguing question: Is this solely due to the prior local disinfection of the puncture site, or does lidocaine, with or without hyaluronan or corticosteroids, potentially exhibit antimicrobial effects?

Our study holds high translational relevance for future clinical research. We aimed to determine whether the addition of local anesthetics like lidocaine to commonly used substances in injection therapy could prevent bacterial growth potentially introduced into the joint during injection, thereby reducing the risk of developing septic arthritis.

## 2. Results

### 2.1. Concentration-Dependent Antimicrobial Effect over 16 h

**Figure 1:**
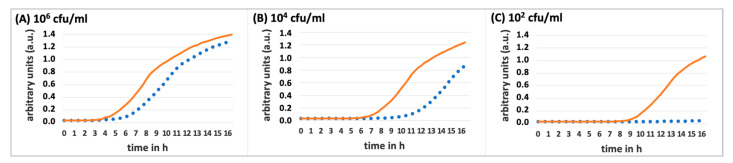
Influence of 1% lidocaine on *S. aureus* growth dynamics with various bacterial densities; This figure shows the extension of the lag phase for *S. aureus* in the presence of 1% lidocaine (dotted lines) compared to the growth control (solid lines) across various bacterial densities in TSB. This effect becomes lower with increasing bacterial density: (**A**) 10^6^ CFU/mL, (**B**) 10^4^ CFU/mL, and (**C**) 10^2^ CFU/mL.

**Figure 2:**
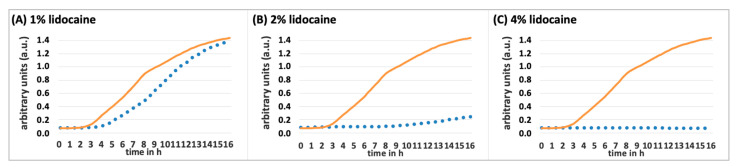
Impact of various lidocaine concentrations on *S. aureus* growth dynamics. This figure illustrates the inhibitory effects of increasing concentrations of lidocaine on the growth of *S. aureus* at 10^6^ CFU/mL. The inhibited growth (dotted lines) is depicted alongside the growth control (solid lines) in TSB, demonstrating greater suppression with higher lidocaine concentrations: (**A**) 1% lidocaine, (**B**) 2% lidocaine, and (**C**) 4% lidocaine.

The growth patterns of *S. aureus* were analyzed in relation to varying concentrations of lidocaine and differing bacterial densities of *S. aureus*, as detailed in Figure 1 and Figure 2.

The data (n = 8) show that lidocaine has an increasing antimicrobial effect at a constant concentration while reducing the bacterial density up to 10^2^ CFU/mL. However, at higher densities of 10^4^ and 10^6^ CFU/mL, the bacteria were able to recover effectively after a prolonged lag phase by 2–3 h (10^6^ CFU/mL) and 4–5 h (10^4^ CFU/mL). At a bacterial density of 10² CFU/mL—within the range of potential contamination introduced during a suboptimally performed intra-articular injection [5]—bacterial growth was nearly completely inhibited, evidenced by an extended lag phase of at least 7 h. After 16 h of incubation, these densities reached optical density values comparable to those of the respective growth controls (at 10^6^ CFU/mL: OD (600 nm) = 1.39 compared to control at 1.48; at 10^4^ CFU/mL: OD (600 nm) = 1.25 compared to the growth control of 1.42). A significant anti-microbial effect of 1% lidocaine was only observed at the lower bacterial density of 10^2^ CFU/mL, where the OD (600 nm) after 16 h was 0.11 compared to the growth control of 1.22.

A 1% lidocaine concentration extends the lag phase by 2 h; however, as shown earlier in Figure 1, the pathogens eventually recover, reaching similar densities to the growth control after 16 h. With 2% lidocaine, the extension of the lag phase is notably, increasing by 6 h and for 4%; one does not observe growth within 16 h compared to the growth control.

### 2.2. Antimicrobial Effect of 1%/2% Lidocaine with Triam40 and Hyaluronan^®^

**Figure 3:**
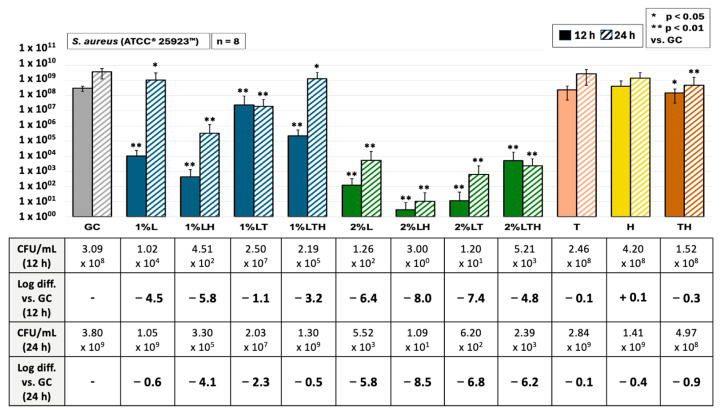
Antimicrobial efficacy of 1% and 2% lidocaine containing test solutions against *S. aureus* over 12 and 24 h. Lidocaine solutions were tested solely (L) or in combination with hyaluronan (LH) or Triam40 (LT) or with both substances (LTH). All test groups referred to unaltered bacterial growth culture under the same conditions (media, volume, temperature, and time of incubation). Moreover, further control groups at same bacterial concentrations were tested without lidocaine in the presence of the mentioned therapeutic additives for joint injections (Triam40, hyaluronan, and both: T, H, TH).

These comprehensive experiments (n = 8) investigate the antimicrobial properties of 1% and 2% lidocaine in different formulations, both alone and in combination with Triam40 and hyaluronan^®^, against *S. aureus,* as presented in Figure 3.

Significant outcomes were achieved for all the tested combinations, except for the pure applications of Triam40 and hyaluronan, across both 12 and 24 h when compared to the growth control. Notably, all combinations involving lidocaine yielded highly significant results (*p* < 0.01) after just 12 h and maintained significant (*p* < 0.05) or highly significant (*p* < 0.01) results after 24 h.

Using 1% lidocaine, lidocaine and hyaluronan displayed the most substantial antimicrobial impact, with log reductions of −5.8 and −4.1 observed at 12 and 24 h. Similarly, among the 2% lidocaine formulations, the pairing of lidocaine with hyaluronan stood out, producing even more pronounced log reductions of −8.0 and −8.5 at 12 and 24 h, respectively. This experiment effectively demonstrated the potent antimicrobial properties of lidocaine across various combinations. It was also observed that pathogens, particularly in the 1% lidocaine pairings, exhibited partial recovery after 24 h. This observation highlights the inhibitory impact of the treatment, which contributes to an extended log phase.

**Figure 4:**
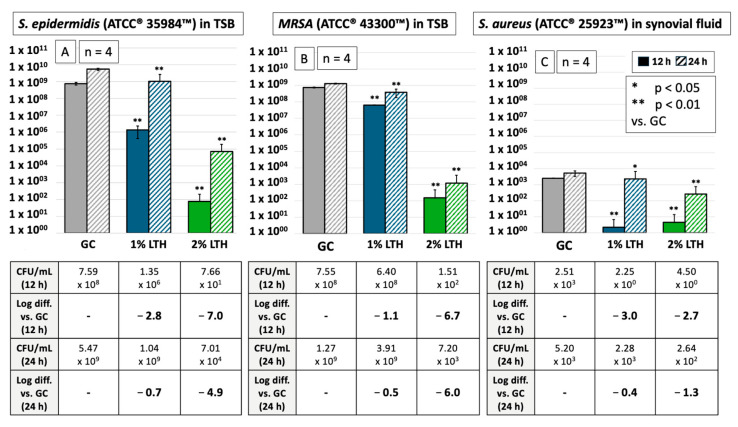
Antimicrobial effects of 1%/2% lidocaine formulations on *S. epidermidis* (**A**) and *MRSA* (**B**) in TSB and on *S. aureus* in synovial fluid (**C**). This figure demonstrates the significant antimicrobial effects of 1% and 2% lidocaine, combined with Triam40 and hyaluronan (LTH), against *S. epidermidis* (filled bars) and against MRSA (checkered bar) in TSB, as well as against *S. aureus* (lined bar) in synovial fluid. These effects are compared to the growth control at an initial bacterial density of 300 CFU/mL over 12 and 24 h. Notably, the 1% lidocaine formulation shows no significant effectiveness after 24 h in synovial fluid.

To determine whether these findings could be applicable to other Gram-positive pathogens or to synovial fluid, additional experiments were conducted. These included testing against 300 CFU/mL MRSA and *S. epidermidis* in TSB, as well as 300 CFU/mL *S. aureus* in synovial fluid (n = 4). The results of these studies are presented in Figure 4.

Comparing the results in Figure 3 and Figure 4 reveals distinct patterns of antimicrobial activity. After 12 h in TSB, 1% lidocaine achieved a higher log reduction against *S. aureus* (−4.5) and *S. epidermidis* (−2.8) compared to *MRSA* (−1.1). However, by 24 h, the reductions were similar across all the tested pathogens in TSB (−0.6, −0.7, and −0.5 respectively). With 2% lidocaine, the effect was consistent for all pathogens at both 12 (−6.4, −7.0, and −6.7) and 24 h (−5.8, −4.9, and −6.0). Significant results were observed for both *S. epidermidis* and *MRSA* after 12 h (*p* < 0.05 and *p* < 0.01, respectively) and 24 h in TSB. These findings underscore again a statistical correlation between increased lidocaine concentrations and enhanced antimicrobial effects; although the bacterial strains exhibited recovery with both lidocaine concentrations, the rebound was less pronounced with the 2% concentration.

For *S. aureus* in synovial fluid, the outcomes diverged significantly from those in TSB; the antimicrobial effects were markedly higher after 12 h, the bacterial density decreased by −3.0 logs for 1% and −2.7 for 2% lidocaine, reducing to less than 5 CFU/mL. By 24 h, however, the antimicrobial effect had significantly diminished, with notably higher concentrations detected (2.28 × 10^3^ CFU/mL for 1% lidocaine and 2.64 × 10^2^ CFU/mL for 2% lidocaine).

Notably, only the 2% lidocaine demonstrated significant antimicrobial activity after 24 h compared to the control in synovial fluid.

## 3. Discussion

Our growth experiments demonstrated that lidocaine exerts a bacteriostatic effect on *S. aureus*, extending the lag phase at a clinically used concentration of 1%, depending on bacterial density. Lidocaine inhibited growth during the first 12 h but did not achieve a bactericidal effect, as the bacteria recovered, and their cell numbers increased after this period. Combinations of 1% and 2% lidocaine with Triam40 and/or hyaluronan significantly reduced bacterial growth at both 12 and 24 h compared to the control in TSB. Reductions in TSB ranged from 1.1 to 8.0 log levels after 12 h and 0.5 to 8.5 log levels after 24 h. In the synovial fluid model, log reductions against *S. aureus* were significantly lower, ranging from −2.7 to −3.1 after 12 h and from −0.4 to −1.3 after 24 h, with reduced growth also observed in the growth control under these conditions.

The antibacterial effect of lidocaine alone is well documented in vitro [17,18,26,27,28,29]. In the study of Parr et al., lidocaine demonstrated a dose-dependent inhibition of growth of *S. aureus*, tested for a concentration of 1%, 2% and 4% lidocaine, among other pathogens [30]. Olsen et al. reported reduced growth of *S. pneumoniae* in culture of bronchoalveolar lavage [31] in the presence of 2% lidocaine. The work of Kesici et al. focused on the combination of lidocaine with the vasopressor agent adrenaline, showing antimicrobial effects of lidocaine both alone and in combination [28]. Interestingly, results in different experimental setups lead to divergent conclusions. Recently, a study published by Tandra et al. [32] stated no preventive effect of lidocaine on bacterial growth in an agar diffusion testing setup. We believe our experimental setup offers a more realistic approximation of clinical conditions, as we opted to test bacterial growth in fluids such as TSB and synovial fluid instead of on agar, better replicating the fluid environment within the knee. On the other hand, Liu et al. documented a pronounced antimicrobial effect of lidocaine and concluded that using infiltration anesthesia prior to diagnostic arthrocentesis for obtaining synovial fluid might lead to false-negative results [33]; conversely, this suggests that lidocaine could serve as a preventive measure against intraarticular bacterial growth and infection.

Our study synthesizes various perspectives on the potential effects of intraarticular lidocaine injections, highlighting its dual role as both a therapeutic agent—with antibacterial and antinoceptive properties—and as a “carrier” for other therapeutic substances. Furthermore, our research underscores lidocaine`s utility as a prophylactic (infection preventing) additive in injections, establishing its comprehensive role in clinical interventions. To date, the evaluation of bacterial growth in synovial fluid with added lidocaine, both lidocaine alone and in combination with Triam40 and hyaluronan, has not been documented in the scientific literature. Depending on the lidocaine concentration, bacterial growth was either slowed (1% and 2% lidocaine) or nearly completely halted (4%) by lidocaine alone, which is in accordance with other workgroups using similar study protocols in vitro. Additionally, our findings revealed an increasing antimicrobial effect of lidocaine at a consistent concentration, accompanied by a reduction in bacterial density. However, for all three bacterial concentrations tested (10^2^, 10^4^ and 10^6^ CFU/mL), the lidocaine concentration of 1% was not sufficient to completely inhibit bacterial growth but to extend the lag phase by 7 h. This implies that the lag phase, where bacterial growth is delayed before the exponential growth phase begins, is significantly extended, or the growth phase is even completely stopped, at lower bacterial densities. Such a change in timing could hypothetically provide eucaryotic organisms the crucial window needed to effectively respond to bacterial invasions. Considering that the bacterial density in the knee following pathogen introduction may be lower than the 300 CFU/mL tested in this study, a 1% concentration of lidocaine could potentially be sufficient to fully prevent any subsequent infection. As shown here, 1% and 2% lidocaine has significantly curtailed the bacterial growth of *Staphylococcus aureus*, *MRSA*, and *Staphylococcus epidermidis*, both alone and in combination with hyaluronan and Triam40 across various media including TSB and synovial fluid after 12 and 24 h. However, the pathogens tested here can recover after 12 h. In our experiments, the antimicrobial effect of lidocaine on *S. aureus* was notably pronounced in synovial fluid and demonstrated minimal growth in synovial control, aligning with the typical clinical scenario within the knee. This observation could potentially reinforce the role of lidocaine in preventing infections in such clinical settings.

Despite its longstanding use, the intraarticular application of local anesthetics has sparked increasingly contentious debates over the past few decades, particularly following instances where post-arthroscopic chondrolysis in the shoulder was causally linked to intra-articular pain pump catheters delivering local anesthetics. This association has been documented in various studies [25,34,35,36]. Subsequently, research in both in vitro settings and animal studies highlighted the cytotoxic effects of local anesthetics on chondrocytes [23,24,25,37,38]. Nonetheless, several in vivo studies have not confirmed these severe detrimental effects on cartilage [39,40], suggesting that the single-dose administration of local anesthetics into the joint may not pose clinically significant harm. However, clinical trials specifically addressing this issue remain rare. Vrachnis et al. described the effects of local anesthetics on histological features of rat cartilage, reporting no macroscopic and histological changes in short- and long-term follow up [41]. This allows for the question of whether the described possible side effects of local anesthesia injections into the joints are of actual clinical relevance for the patients [23] and whether cell culture, which has most widely been used for quantifying effects of local anesthetics on chondrocytes, is really the most suitable method. Future clinical trials collecting long term data on history of patients with osteoarthritis undergoing single or multiple injections of local anesthetics during conservative therapy would be of great value.

Intra-articular injections into arthroplasty joints are controversial and generally discouraged, especially with corticosteroids, because of a possible increased risk of prosthetic joint infection and revision [42,43]. The biologic activity of steroids may have a negative immunomodulatory effect on the intra-articular environment [44], making a possible inoculation of bacteria during the process of injection even more dangerous. The presence of implants itself seems to foster bacterial growth, and avascular surfaces promote biofilm formation, allowing bacteria to escape cellular defense mechanisms [45]. Yet, for patients with persistent pain after arthroplasty and unknown etiology, it might be a conservative treatment option before performing revision arthroplasty. Our results strongly suggest that lidocaine, even in combination with corticosteroids, has a potent antimicrobial effect. The critical view of intra-articular steroid injections in arthroplasty joints should therefore be reconsidered, as the addition of lidocaine or other local anesthetics as a “carrier” for glucocorticoid injection might reduce the risk of iatrogenic infection and thus override the expected negative effects. The combination of the antinociceptive and antimicrobial effects of lidocaine with the anti-inflammatory effects of corticosteroids may be particularly beneficial in joint arthroplasty patients who have had infection ruled out but have persistent pain or are medically ineligible for revision arthroplasty. On further reflection, another implication may be the use of intra-articular local anesthetics (both alone and in combination with other local antibiotic delivery options [46,47,48]) in the conservative management of chronic paucibacillary infections after joint arthroplasty caused by low virulence pathogens, namely low-grade infections [49]. Further clinical research is needed to verify this hypothesis.

### Limitations of the Study

One limitation of our study is its in vitro design. For example, it can be assumed that lidocaine is diluted further in vivo by synovial fluid, thus making it harder to predict possible effects.

In addition, the synovial fluid was sterilized using a cellulose filter to remove possible pathogens. It can be assumed that cells, proteins, and other bioactive molecules were also reduced to a certain extent, which is why interactions between synovial fluid and lidocaine, or bacteria may well be altered at a molecular level. Therefore, the data found here cannot be fully transferred to in vivo conditions but provide an initial indication in a synovial fluid-like environment. For this, more ex vivo and in vivo tests will be needed in the future.

The optical density measurement for observing the concentration-dependent growth processes is also subject to typical method limitations. Even if in principle no direct distinction can be made here between living/dead cells and photometers usually only have linear measuring ranges between OD 0.1 and 0.6, conclusions can still be drawn about the antimicrobial effect of lidocaine based on the dynamic observation. The comparison of the growth kinetics (increase in cell density and duration of the lag phase) with and without the effect of lidocaine allows interpretations to be made in this respect, as this is largely dependent on the proportion of vital cells.

Furthermore, only ATCC strains were used in these experiments. Although these are clinical isolates, future investigations could include additional testing with wild-type strains directly obtained from patients.

Still, the experimental setup using different combinations of fluid allows for the precise evaluation of the concentration-dependent effects of lidocaine on bacterial growth and the possible influence of commonly used combinations of lidocaine and other intraarticular injectables better than, e.g., agar diffusion settings.

Another limitation comprises the fact that we did not address the pathways of possible chondrotoxic effects of lidocaine, so further research is necessary to quantify these effects. To put it in other words: it will be of great interest to compare the possible risks (and to find ways of eliminating these risks) of a singular injection into an arthritic joint aiming at disrupting inflammatory processes to those of accepting prolonged inflammatory processes in that very joint.

## 4. Materials and Methods

### 4.1. Concentration-Dependent Antimicrobial Effect over 16 h

Initially, the effect of 1% lidocaine (lidocaine-hcl, Sigma Aldrich, St. Louis, MO, USA, article no. L5647) on the growth behavior of different *S. aureus* (ATCC^®^ 25923™) concentrations with 10^2^ CFU/mL, 10^4^ CFU/mL, and 10^6^ CFU/mL in TSB (tryptic soy broth, Sigma Aldrich, St. Louis, MO, USA, article no. 22092) was investigated. The different growth curves were considered at 10^2^, 10^4^, and 10^6^ CFU/mL to investigate the concentration-dependent antimicrobial effect.

However, only concentration ranges around 100 CFU/mL have clinical relevance regarding the problem of the cause of the injection under consideration; pathogen densities of 10^4^ and 10^6^ would already correspond to florid joint infections but were also tested for the basic investigation of the influence of the pathogen quantities on the lidocaine quantities.

For this purpose, on the first day of the experiment, a colony of the *S. aureus* strain was transferred from the agar plate into 30 mL of TSB and incubated overnight in a shaking incubator at 37 °C to ensure the presence of viable pathogens the next day. On the following day, a 1% lidocaine solution was prepared by dissolving 0.075 g lidocaine in 7.5 mL TSB and homogenizing the mixture through vortexing. From both the pure TSB and the 1% lidocaine solution, 6480 µL each was transferred into three rows of 8 wells with 270 µL per well on a 96-well plate. The overnight incubated bacterial suspension was diluted with an Eppendorf photometer (Eppendorf, Wesseling-Berzdorf, Germany) in TSB to an OD (600 nm) = 0.1, which corresponds to a bacterial density of 10^8^ CFU/mL according to our calibration curve. To obtain three approaches with defined bacterial concentrations, the bacterial suspension was diluted in a ratio of 1:10 (10^7^ CFU/mL), 1:1000 (10^5^ CFU/mL), and 1:10,000 (10^3^ CFU/mL). To confirm the targeted bacterial densities, serial dilutions of all bacterial suspensions were prepared in PBS. Each dilution level was plated with 100 µL, and after incubation at 37 °C overnight, the colonies were quantified the following day. A total of 480 µL from each of the three bacterial suspensions was added to the row with pure TSB (growth control) and to the rows containing the lidocaine solution (samples), with 30 µL dispensed per well. This step further diluted the bacterial densities by a factor of 10, resulting in final concentrations of 10², 10⁴, and 10⁶ CFU/mL. The growth dynamics of the individual samples (n = 8) were continuously monitored using a UV/VIS spectrometer (Multiskan Go, Thermo Fisher Scientific, Darmstadt, Germany) over 16 h at 37 °C. The results were graphically represented as growth curves to highlight any variations between the lidocaine-treated samples and the control.

To observe the growth pattern of this *S. aureus* strain with a density of 10^6^ CFU/mL under the influence of 1%, 2%, and 4% lidocaine, a pre-culture in TSB was first set up as described above and incubated overnight in a shaking incubator at 37 °C. To prepare the lidocaine solutions, 3 mL of TSB was mixed with 0.03 g (1%), 0.06 g (2%), and 0.12 g (4%) of lidocaine and homogenized by vortexing. In a 96-well plate, one row of 8 wells each was filled with pure TSB, 1%, 2%, and 4% lidocaine solutions at 270 µL per well. The bacterial suspension was again diluted to an OD (600 nm) = 0.1 (10^8^ CFU/mL) and further diluted 1:10 with pure TSB to achieve a bacterial density of 10^7^ CFU/mL, which was verified by dilution series, plating 100 µL of each dilution steps on agar and quantifying after overnight incubation at 37 °C. Both the wells with pure TSB (growth control) and those with the different lidocaine solutions (samples) were inoculated with 30 µL of the bacterial suspension per well. The growth dynamics of the individual samples (n = 8) were also continuously monitored at 37 °C over a 16 h period, and the results were graphically represented as growth curves as described before.

### 4.2. Antimicrobial Effect of 1%/2% Lidocaine with Triam40 and Hyaluronan

Moreover, the antimicrobial effects of lidocaine at concentrations of 1% and 2%, both alone and in combination with hyaluronan (Ostenil^®^, TRB Chemedica, Geneva, Switzerland, AG, PZN: 08896935) or crystalline corticoids (Triam40, Zentiva Pharma GmbH, Prague, Czech Republic, PZN: 06155270), against *S. aureus*—the primary pathogen implicated in septic arthritis—were explored by this study. These effects were assessed in both TSB (n = 8) and synovia fluid (n = 4). Additionally, the efficacy of these combinations was also investigated against *S. epidermidis* (ATCC^®^ 35984™) and methicillin-resistant *S. aureus* (ATCC^®^ 43300™) in TSB (n = 4).

On the next day, various sample liquids, as outlined in Table 1 and Table 2, were prepared by mixing and vortexing homogenously. For experiments involving synovial fluid, knee joint fluids from three patients with aseptic osteoarthritic joint inflammation, aged between 60 and 70 years, were pooled, sterile filtered by a 0.22 μm filter (Sartorius, Göttingen, Germany, 16534 K), and stored in the freezer for one week prior to use (Ethics vote no. 2019-30_1-S-SSR from the TUM University hospital).

Each bacterial strain was pre-incubated as a single colony in 30 mL TSB overnight at 37 °C for 24 h before testing and diluted to an OD value of 0.1 (10^8^ CFU/mL). The bacterial suspension was initially diluted through a serial dilution (1:10) over two steps in PBS, resulting in a 100-fold reduction, and subsequently diluted at a 1:333 ratio by mixing 150 µL of the second dilution step with PBS to a total volume of 50 mL, followed by vortexing. Subsequently, 444 µL was mixed with 4 mL (*test 1,* Table 1) and 222 µL with 2 mL (*test 2,* Table 2), vortexed, and further diluted 1:10 to achieve a final bacterial density of 300 CFU/mL. Immediately after preparation of all samples, 100 µL of each sample was plated on CBA plates to verify the initial bacterial density following overnight incubation at 37 °C. The samples were then incubated in 50 mL centrifuge tubes at 130 rpm in a shaking incubator at 37 °C for 24 h. At 12 and 24 h, dilution series with 12 steps each (dilution 1:10) were prepared by mixing 30 µL of the sample with 270 µL of PBS. Subsequently, 100 µL of each dilution step was plated on CBA plates and incubated for 24 h at 37 °C for quantitative analysis on the following day. The resulting bacterial counts were converted to CFU/mL and statistically analyzed using a t-test to compare the growth of each sample against the control (bacteria in TSB), with significance levels noted as *p* < 0.05 (*), *p* < 0.01 (**) and *p* < 0.001 (***).

### 4.3. Additional Information: Use of AI-Assisted Text Processing

To support the development of parts of the manuscript, particularly in the formulation of scientific texts, the AI language model ChatGPT (Version: GPT-4, OpenAI) was used. The model served as a tool to generate and refine text based on scientific guidelines and existing data. Sections drafted by ChatGPT were subsequently reviewed, scientifically validated, and adjusted by the authors as necessary to ensure scientific accuracy and clarity. The use of ChatGPT was carried out in accordance with ethical guidelines, and the authors take full responsibility for the final content of the manuscript.

## 5. Conclusions

The present study shows an anti-infective dose-dependent effect of lidocaine on bacteria count in different fluids and combinations of injectables due to an extended lag-phase of bacterial growth. The study underlines the reported antimicrobial effects of lidocaine and extends systematic knowledge about these effects in a synovial fluid-like environment and when combined with hyaluronan and triamcinolone.

This might explain the low infection incidence of septic arthritis after therapeutic injections into joints and may possibly play a major role in preventing clinical complications in clinical practice. As negative effects of local anesthetics on chondrocytes have been reported, conservative treatment of osteoarthritis remains highly individual. Further research is necessary to clarify the actual clinical relevance of these effects and to determine the optimal administration form and the optimal concentration of intraarticular lidocaine, as well as the optimal timing in a multimodal therapy concept.

## Figures and Tables

**Table 1:** Sample compositions for testing the antimicrobial effect of lidocaine 1%/2%, both as standalone agent and in combination with hyaluronan and Triam40 in TSB.

Type of Sample	Acronym	TSB (mL)	Lidocaine (mg)	Triam40 (mL)	Hyaluronan (mL)	PBS (mL)	BS * (mL)	Sample Volume (mL)
**lidocaine 1%**	**L1**	2.5	25	-	-	1.5	0.444	4.44
**lidocaine 2%**	**L2**		50	-	-	1.5		
**Triam40**	**T**		-	0.5	-	1.0		
**hyaluronan**	**H**		-	-	1	0.5		
**Triam40 + hyaluronan**	**TH**		-	0.5	1	-		
**lidocaine 1% + hyaluronan**	**L1H**		25	-	1	0.5		
**lidocaine 1% + Triam40**	**L1T**		25	0.5	-	1.0		
**lidocaine 1% + Triam40 + hyaluronan**	**L1TH**		25	0.5	1	-		
**lidocaine 2% + hyaluronan**	**L2H**		50	-	1	0.5		
**lidocaine 2% + Triam40**	**L2T**		50	0.5	-	1.0		
**lidocaine 2% + Triam40 + hyaluronan**	**L2TH**		50	0.5	1	-		
**growth control (bacteria in TSB)**	**GC**		-	-	-	1.5		

BS *: bacterial inoculum with defined bacterial density to reach standardized test samples at 300 CFU/mL.

**Table 2:** Samples prepared for testing the antimicrobial effect of lidocaine 1%/2% with hyaluronan and Triam40 in synovia.

Type of Sample	Acronym	Synovia (mL)	Lidocaine (mg)	Triam40 (mL)	Hyaluronan (mL)	PBS (mL)	BS ** (mL)	Sample Volume (mL)
**lidocaine 1% + Triam40 + hyaluronan**	**L1%THSyn**	1.25	12.5	0.25	0.5	-	0.222	2.22
**lidocaine 2% + Triam40 + hyaluronan**	**L2%THSyn**		25	0.25	0.5	-		
**growth control (bacteria in synovia)**	**GCSyn**		-	-	-	0.75		

BS **: bacterial inoculum with defined higher bacterial density to reach standardized test samples at 300 CFU/mL in 2.22 mL.

## Data Availability

All data associated with this study are present in the paper. More data supporting this study’s findings are available from the corresponding author on request.
